# Combination of PET and Magnetoencephalography in the Presurgical Assessment of MRI-Negative Epilepsy

**DOI:** 10.3389/fneur.2013.00188

**Published:** 2013-11-21

**Authors:** Sylvain Rheims, Julien Jung, Philippe Ryvlin

**Affiliations:** ^1^Department of Functional Neurology and Epileptology, Institute of Epilepsies (IDEE), Hospices Civils de Lyon, Lyon, France; ^2^INSERM U1028/CNRS UMR5292, Lyon Neuroscience Research Center, Lyon, France

**Keywords:** PET, magnetoencephalography, partial epilepsy, epilepsy surgery

## Abstract

Despite major advances in neuroimaging, no lesion is visualized on MRI in up to a quarter of patients with drug-resistant focal epilepsy presenting for presurgical evaluation. These patients demonstrate poorer surgical outcomes than those with lesion seen on MRI. Accurate localization of the seizure onset zone (SOZ) is more difficult in MRI-negative patients and often requires invasive EEG recordings. Positron emission tomography (PET) and magnetoencephalography (MEG) have been proposed as clinically relevant tools to localize the SOZ prior to intracranial EEG recordings. However, there is no consensus regarding the optimal gold standard that should be used for assessing the performance of these presurgical investigations. Here, we review the current knowledge concerning the usefulness of PET and MEG for presurgical assessment of MRI-negative epilepsy. Beyond the individual diagnostic performance of MEG and of different PET tracers, including [^18^F]-fluorodeoxyglucose, [^11^C]flumazenil, and markers of 5-HT1A receptors, recent data suggest that the combination of PET and MEG might provide greater sensitivity and specificity than that of each of the two individual tests in patients with normal MRI.

## Introduction

Patients’ selection for epilepsy surgery is a two-step procedure that first aims to identify potential candidates for presurgical evaluation, and then to determine in each assessed individual whether benefit:risk ratio for surgery is acceptable (1). This process primarily requires localizing the seizure onset zone (SOZ), i.e., the minimum amount of brain tissue that should be resected to render the patient seizure-free. The identification of the SOZ results from the integration of a set of arguments, particularly electro-clinical data obtained during long-term video-EEG monitoring and results of optimal high-resolution brain MRI (1). Identification of a structural abnormality on brain MRI is of paramount importance, since such abnormality usually represents the core of the presurgical and surgical strategy. Post-operative seizure outcome positively correlates with the presence of an epileptogenic brain lesion on MRI (2). Despite major advances in neuroimaging, MRI remains negative in up to a quarter of patients presenting for presurgical evaluation (3). Poorer surgical outcome in patients with MRI-negative partial epilepsy partly reflects the difficulties for determining the exact localization and extension of the SOZ. In these patients, intracranial EEG recording is usually mandatory to ensure the delineation of the SOZ. Whatever the invasive recording technique used, subdural electrodes or stereoelectroencephalography (SEEG), brain sampling remains limited by safety issues. The placement of subdural or depth electrodes should therefore be individualized according to the most possibly precise and reliable hypothesis regarding the localization of the SOZ. Overall, the outcome of invasive EEG monitoring primarily depends on the quality and interpretation of the non-invasive data used to decide of the brain regions to be targeted.

Positron emission tomography (PET) and magnetic source imaging (MSI) have been proposed as valuable tools to help localizing the SOZ prior to intracranial EEG recordings. However, the comparative performance of these presurgical investigations remains controversial, especially in patients with MRI-negative epilepsy. On the other hand, recent data suggest that PET and magnetoencephalography (MEG) might be complementary, with their combination yielding increased sensitivity and specificity as compared to each test used in isolation. Here, we review the current knowledge concerning the usefulness of PET and MEG for presurgical assessment of MRI-negative epilepsy and discuss the impact of combining their results on SOZ localization.

## PET Studies in MRI-Negative Focal Epilepsy

Several PET tracers have been evaluated as potential markers of the SOZ in patients with epilepsy. [^18^F]-fluorodeoxyglucose ([^18^F]-FDG), which brain accumulation reflects the local cerebral metabolic rate of glucose, is the most widely used tracer in patients with epilepsy (4). However, multiples other tracers that target neurotransmitter systems have also been studied in epilepsy, primarily those labeling GABA_A_ and 5-HT1A receptors (1).

Before discussing the individual diagnostic performance of these tracers, some general methodological points should be underscored: (i) whatever the radiotracer used, PET data are usually obtained during the interictal period. Although programing ictal PET is feasible (5), its constraints, related to radioligands kinetics and images acquisition, usually overcome its potential benefit in daily practice. Possibility of seizure occurrence during “interictal” PET acquisition should however be taken into account since this might result in false lateralization of PET abnormality; (ii) PET findings should be assessed with reference to structural imaging, especially in patients with normal MRI. Indeed, PET/MRI coregistration allows to more easily distinguish true hypometabolism from the sole impact of focal atrophy or wide sulcus, and to detect subtle MRI abnormalities which would not have been recognized otherwise. It has been shown that [^18^F]-FDG-PET/MRI coregistration in patients with focal cortical dysplasia (FCD) improved detection of focal metabolic abnormalities by 35–40%, both in patients with abnormal and normal MRI (6). A study in children with MRI-negative epilepsy also reported that coregistered [^18^F]-FDG-PET/MRI data resulted in the detection of subtle MRI abnormalities not previously recognized in 9 out 31 patients (7); (iii) interpretation of PET images primarily relies on standard visual analysis, but statistical analysis, with the widely used Statistical Parametric Mapping (SPM) software, might improve the diagnostic yield of PET. Over the past years, some authors have thus reported that the use of SPM can result in greater sensitivity and specificity of PET imaging in patients with MRI-negative epilepsy (8, 9). However, other studies did not confirm this finding (10). In addition, various methodological pitfalls of SPM analyses should be kept in mind, including artifacts of various origins, presence of abnormalities in some control subjects (11), and age difference between patients and controls (12). In our clinical experience of more than 300 FDG-PET where both visual and SPM analyses were performed, the latter proved useful in two situations: (i) detecting subtle hypometabolism over the mesial frontal or parietal regions, where spill-over and partial volume effects often result in bilateral and symmetrical abnormalities difficult to capture with visual analysis and (ii) demonstrating the area of maximum hypometabolism in patients with multiple or extensive visually detected abnormalities. Improvement in the performance of SPM findings can be obtained by running such voxel-based analysis on maps of asymmetry index between the two hemispheres, rather than on the raw data (13).

### [^18^F]-FDG-PET

Hypometabolism on interictal [^18^F]-FDG-PET is a hallmark of the SOZ as well as surrounding areas (4). Several studies have evaluated the sensitivity and specificity of [^18^F]-FDG-PET in patients with MRI-negative focal epilepsy (see Table 1).

**Table 1:** **Diagnostic accuracy of [^18^F]-FDG-PET in MRI-negative focal epilepsy**.

Study	Epilepsy	Age group	Number of patients	Abnormal PET findings (%)	Specificity of PET abnormalities		Remarks
					Concordant lateralization with the SOZ (%)	Focal hypometabolism concordant with the localization of SOZ (%)	
Juhasz et al. (14)	T, ET	Children and adults	9	100	100	11	–
O’Brien et al. (15)	T, ET	Adults	36	–	–	69.4	–
Hong et al. (16)	T, ET	Children and adults	28	57.1	57.1	42.9	–
Carne et al. (17)	T	Adults	30	–	86.7	33.3	–
Chapman et al. (18)	T, ET	Children and adults	24	–	66.7	66.7	–
Lee et al. (19)	ET	Children and adults	79	–	44.3	–	–
Bien et al. (20)	T, ET	Adults	17	58.8	–	29.4	–
Jayakar et al. (21)	T, ET	Children	10	100	–	60	–
Knowlton et al. (22, 23)	T, ET	Children and adults	64	85.2	–	29	–
Brodbeck et al. (24)	T, ET	Children and adults	9	77.8	77.8	55.5	–
Immonen et al. (25)	T	Adults	17	–	58.8	–	–
Rubi et al. (7)	ET	Children	31	67.7	58.1	32.2	–
Seo et al. (26)	ET	Children	14	100	71.4	21.4	–
Chassoux et al. (10)	T, ET	Children and adults	23	95.6	95.6	59.1	Taylor-type FCD
LoPinto-Khoury et al. (27)	T	Adults	46	100	100	–	All included patients had PET+/MRI−
Gok et al. (28)	T	Adults	38	–	84.1	6.7	–

T, temporal lobe epilepsy; ET, extra-temporal epilepsy; SOZ, seizure onset zone.

[^18^F]-FDG-PET abnormalities were observed in a majority of patients, both in adults and children. In the largest cohorts, abnormal PET was reported in 53–100% of patients (7, 12, 17, 20, 22, 27). However, the congruence between PET findings and the SOZ localization was generally low. Although abnormalities usually showed concordant lateralization with the SOZ, non-localizing multilobar abnormalities, or falsely localizing abnormalities were reported in up to 50% of patients (7, 16, 19, 20, 22, 29). Furthermore, in patients with focal abnormality, the hypometabolic area was frequently more widespread than the SOZ (7, 14, 16, 17, 26), although remote abnormalities appeared to be related to the location of the primary seizure focus (14, 17). In a cohort of 61 patients with temporal or extra-temporal MRI-negative epilepsy, where intracranial EEG was used as a gold standard to localize the SOZ, [^18^F]-FDG-PET sensitivity, and specificity were 39.5% (95% CI: 31.4–47.4) and 53.3% (95% CI: 30.0–76.0), respectively (22). When the resected area of the 51 patients with Engel class I surgical outcome served as a reference for defining the SOZ, [^18^F]-FDG-PET sensitivity, and specificity increased to 59% (95% CI: 47.4–67.4) and 79% (95% CI: 58.7–92.5), respectively (23). The concordance between [^18^F]-FDG-PET data and the SOZ was particularly low in patients with extra-temporal SOZ. Accordingly, sensitivity of [^18^F]-FDG-PET to detect focal hypometabolism concordant with the SOZ and to predict seizure outcome was <40% in the majority of studies which evaluated patients with extra-temporal epilepsy (7, 8, 16, 22, 26). However, two situations, in which the diagnostic accuracy of [^18^F]-FDG-PET in patients with MRI-negative epilepsy might be higher, should be individualized: (i) patients with mesial temporal lobe epilepsy without evidence of hippocampal sclerosis on MRI; and (ii) patients with MRI-negative Taylor-type focal cortical dysplasia.

In a series of 30 patients with MRI-negative temporal lobe epilepsy, [^18^F]-FDG-PET showed abnormalities concordant with SOZ localization in 26 patients (87%) (17). Among the subgroup of 17 patients with focal hypometabolism on [^18^F]-FDG-PET who had undergone temporal lobe surgery, 15 (88%) were seizure-free after a mean post-operative follow-up of 38 months, a surgical outcome similar to that observed in a control group that included patients with hippocampal sclerosis on MRI (17). Interestingly, the authors individualized some clinical differences between the two patient’s groups. Those with MRI-negative PET-positive temporal lobe epilepsy demonstrated lower frequency of history of febrile convulsions and of hippocampal sclerosis at pathological examination, but more widespread hypometabolism than those with hippocampal sclerosis on MRI (17). In a larger series of 46 patients with MRI-negative TLE and unilateral temporal hypometabolism on PET, 5-years seizure-free rate was 75%, versus 78% in the 147 patients with MRI signs of mesial temporal sclerosis (27).

Among patients with extra-temporal MRI-negative epilepsy, those with MRI-occult FCD demonstrate better post-operative seizure outcome than those with normal pathological examination of the surgical specimen (2, 30). Whether or not detected on MRI, Taylor-type FCD are typically associated with clearcut focal hypometabolism on [^18^F]-FDG-PET at the site of the malformation (10, 31, 32). In a recent study of 23 patients with histologically proven Taylor-type FCD and negative MRI, [^18^F]-FDG-PET disclosed a focal or regional hypometabolism in 22 patients (96%), 20 of whom were seizure-free post-operatively (10). Correlation with SEEG data in 20 patients showed that hypometabolic zones restricted to a single gyrus usually matched with the underlying epileptogenic FCD, whereas larger hypometabolic areas included the epileptogenic FCD but also cortical regions not involved at seizure onset. However, the detection of small hypometabolic areas associated with MRI-occult FCD can be challenging in some of the typical locations of such FCD, such as the bottom of the F1–F2 sulcus, if not appropriately oriented by electro-clinical data.

Overall, MRI-negative patients can be separated into two groups: (1) those with TLE or MRI-occult Taylor-type FCD where [^18^F]-FDG-PET demonstrates high sensitivity and specificity associated with excellent surgical outcome and (2) those with other types of MRI-negative drug-resistant partial epilepsy where both [^18^F]-FDG-PET and surgery proved less effective. This raises the issue as to whether normal or not clearly localizing [^18^F]-FDG-PET (i.e., multifocal or multilobar hypometabolism with unclear borders) should be interpreted as a reliable indicator of a very poor surgical prognosis, rather than as a limitation of PET.

### Radioligands of receptors

#### Imaging of GABA_A_ receptors

^11^C-flumazenil ([^11^C]-FMZ) is a selective antagonist of GABA_A_ – benzodiazepine (BZD) receptors (4). The BZD receptors labeled with [^11^C]-FMZ represent modulator sites of the GABA_A_ receptors, which are primarily expressed post-synaptically on the apical dendrites of neurons. A reduced FMZ binding, as observed in patients with partial epilepsies, is thought to primarily reflect an underlying neuronal loss. However, it has been shown that [^11^C]-FMZ PET can demonstrated transient and falsely lateralizing asymmetries, possibly reflecting seizure-related short-term plasticity of BZD receptors (33). In a test-re-test [^11^C]-FMZ PET study, significant seizure-related variations were observed in 5 out of 10 patients, including all three with MRI-negative temporal lobe epilepsy (34).

Several studies have evaluated the clinical utility of [^11^C]-FMZ in patients with MRI-negative focal epilepsy. Most studies evaluated [^11^C]-FMZ PET as a marker of the SOZ and compared it with [^18^F]-FDG-PET. In 2004, a review of the literature identified 45 patients with TLE and a normal MRI who had undergone [^11^C]-FMZ PET (35). [^11^C]-FMZ PET was abnormal in 38 patients (84%), but these abnormalities were considered as surgically informative in only 21 of them (47%). Similar results were observed in patients with extra-temporal epilepsy where [^11^C]-FMZ PET detected abnormalities in 73 out of 102 retrieved cases, including 52 which were judged as clinically relevant (35). Some studies suggested that [^11^C]-FMZ PET sensitivity and specificity might be greater than that of [^18^F]-FDG-PET in patients with MRI-negative epilepsy of both temporal and extra-temporal origin (14, 36, 37). However, other studies did not confirm these findings (29, 38). [^11^C]-FMZ PET has also been proposed to detect subtle MRI-occult cortical microdysgenesis, specifically heterotopic neurons within the periventricular white matter (39–41). However, it should be noted that most [^11^C]-FMZ PET studies in patients with MRI-negative epilepsies were performed more than 10 years ago, using less optimal MRI investigations than those currently available, raising the possibility that subtle MRI abnormalities might have been overlooked. One exception is a recent study that has used [^18^F]-FMZ instead of [^11^C]-FMZ, and reported correct localization of [^18^F]-FMZ abnormalities in 42% of patients with normal MRI, in comparison with 58.8% with [^18^F]-FDG-PET (42).

#### Imaging of 5-HT1A receptors

Three different 5-HT_1A_ receptors antagonist have been used in epilepsy: [^11^C]WAY 100635, [^18^F]FCWAY, and [^18^F]MPPF (11). PET studies with either of these ligands showed a high level of binding in limbic (hippocampus, amygdala, parahippocampal gyrus) and paralimbic (temporal pole, insula, anterior, and posterior cingulate gyri) regions as compared with other neocortical areas (43). In patients with mesial TLE, decreased binding of all three tested tracers was consistently observed within the epileptogenic mesial temporal structures (4), with correlation between the degree of binding reduction and epileptogenicity as evaluated by intracranial EEG recording (44).

A limited number of patients with MRI-negative temporal lobe epilepsy have been investigated with 5-HT1A receptors PET. The studies evaluating [^18^F]MPPF in patients with temporal lobe epilepsy included a total of 10 patients with normal MRI, including nine with mesial temporal SOZ and one with neocortical temporal seizure focus (45, 46). Among the nine patients with a mesial SOZ, seven showed decreased [^18^F]MPPF within the epileptogenic limbic structure, including one with normal [^18^F]-FDG-PET (45, 46). Voxel-based analysis of asymmetry index maps increased the specificity of [^18^F]MPPF abnormalities (13).

Similar data were reported with both [^18^F]FCWAY and [^11^C]WAY 100635. [^18^F]FCWAY has been evaluated in 12 patients with MRI-negative TLE, nine of whom showed congruent abnormalities (75%) with intracranial EEG data or surgical outcome, with a slightly better sensitivity than that of [^18^F]-FDG-PET (47). Another six patients with MRI-negative epilepsy were evaluated with [^11^C]WAY 100635. All showed decreased ipsilateral mesial temporal binding potential of [^11^C]WAY 100635 (48), including four with unremarkable [^18^F]-FDG-PET (48).

Overall, these studies suggest that PET study of 5-HT_1A_ receptors might be of interest in identifying or confirming temporal lobe SOZ, particularly when other imaging modalities, including [^18^F]-FDG-PET, are non-conclusive. Whether or not 5-HT_1A_ receptor PET might have an added value in patients with epilepsy of extra-temporal origin remains to be investigated.

#### Other tracers

Among the other PET tracers evaluated in epilepsy, [^11^C]alpha-methyl-tryptophan (AMT) has a specific relevance in tuberous sclerosis, by showing increased AMT uptake within the epileptogenic tuber, selectively (49). However, this pattern is only observed in about half of patients in the majority of series, and in an even lower proportion of patients in our personal experience (50). Some studies have also shown abnormal AMT asymmetry or increased AMT binding in the presumed SOZ in patients with MRI-negative epilepsy, including a few cases with non-localizing [^18^F]-FDG-PET (51– 53).

## MSI Studies in MRI-Negative Focal Epilepsy

### MSI basics

Source localization of epileptic brain signals (such as epileptic spikes, fast oscillatory signals, or focal background slowing) can be used in the interictal period or during seizures using MEG. Source modeling of those signals and subsequent coregistration on individual brain MRI is referred to as MSI.

During the last 40 years, MEG instruments have evolved from a single-channel portable system to the modern whole head systems with more than 300 channels that are housed in multilayered shielded rooms. Moreover, validated source modeling procedures are now available in several commercial softwares, so that routine clinical use of MSI is now feasible (54–56).

Magnetic source imaging of epileptic spikes require several sequential steps (55, 57, 58): (i) recording of pathological signals with MEG; (ii) preprocessing of MEG data (artifact detection and removal, filtering, visual, or automatic detection of epileptic spikes, averaging or not of epileptic spikes to increase signal-to-noise ratio); (iii) modeling the conduction volumes of the brain using brain MRI tissue segmentation (spherical head models or more realistic head models which take into account individual brain geometry and possible conduction anisotropies between different brain elements); (iv) resolving the so-called inverse problem (i.e., determining the exact location of the neural source(s) best accounting for the recorded MEG data); and (v) co-registrating anatomical MRI data and functional data (brain sources of epileptic spikes). The choice of the conduction and inverse models is important as both solutions have a potential impact on MSI results (59, 60).

Inverse models can be divided into three families. Most clinical studies have used only the first family: classical single or multiple dipole modeling in which a current dipole source model is fitted to the MEG data. Parameters that have to be estimated are the dipole position and strength. The second and third families have been used much less. In the second family [example of methods: MUSIC (61), beamforming (62)], a source is found by scanning all possible positions in the brain. These methods require assumptions about which part of the measured data is signal and which is background or noise. The third family [examples of methods: LORETA (63) or MEM (64)], consists of linear inverse methods in which the data are modeled with only the source strengths as parameters for a distributed set of dipoles at known locations. Assumptions about or constraints on source strength are required for these methods.

The theoretical background of each inverse model is well known but studies comparing the location accuracy of all methods in clinical setting are lacking. Recently, de Gooijer-van de Groep et al. (65) showed that three well known inverse methods (MUSIC, SAMg2, and LORETA) had approximately the same localization accuracy, but that their sensitivity depended on the anatomical location of spike sources. The authors advised to use a combination of those methods in clinical practice.

Magnetic fields recorded by MEG are almost uninfluenced by variation in tissue conductivities (55, 66). Thus, simple models of the volume conductor for MEG source analysis offer high spatial resolution with only minimal distortion (60). In contrast, EEG recordings are more sensitive to volume currents. This explains why more complex models, derived from individual anatomy, are needed for optimal EEG source analysis, and why the latter is more prone to localization errors due to inaccurate segmentation of the brain compartments associated with distinct conductivity. This is especially relevant for patients with large brain lesions or malformations, and those who have undergone prior brain surgery. MEG also suffers limitations, being insensitive to exclusively radially oriented sources, such as those found at the depth of sulci or top of gyri (55, 66). As a consequence, activity from radial sources may be overlooked, while those from oblique source will suffer reduction in amplitude.

Magnetoencephalography benefit from high temporal resolution of less than a millisecond and, under optimal circumstances, spatial resolution of several millimeters (66). However, precisely estimating the accuracy of MSI localization would require reference information as to the actual source location of epileptic paroxysmal transients, which is seldom the case. The degree of inaccuracy may be calculated directly by modeling simulated data from known sources, or alternatively by creating current dipoles between two adjacent intracerebral electrodes *in vivo*. A mean localization error of about 10 mm may be accepted, but this can vary from a few millimeters to as much as a few centimeters. Indeed it is generally well accepted that the localization error: (i) is larger when the source is deeply located; (ii) is smaller when realistic head models are used, compared with spherical models; (iii) is smaller when the spatial sampling of MEG captors is increased; and (iv) decreases when the signal–noise ratio increases (66).

### The diagnostic yield of MSI in MRI-negative patients

Evaluating the diagnostic yield of MSI requires to evaluate the sensitivity of the method and its accuracy. During the last 15 years, numerous studies have been performed to evaluate the sensitivity of MEG for: (a) the detection of epileptic spikes and (b) the detection of localized and focal sources of spikes (see Table 1). The vast majority of those studies have been performed in selected groups of patients referred for MEG evaluation because scalp-EEG disclosed frequent interictal spikes. Therefore, the sensitivity of MSI in the general population is not known. Moreover, very few of these studies specifically investigated MSI in negative MRI patients.

Based on existing studies, the sensitivity of MSI for the detection of focal sources of spikes is estimated between 35 and 90% (67–69). The wide range of sensitivity estimates is partly due to the different methodological approaches used to determine the spatial extent of spike sources. Most studies used spatial clustering of spikes modeled with single dipole models as an estimate of the volumetric extent of spiking volumes (26, 68–70). However, the extent of spikes’ cluster remains difficult to interpret since it depends on multiple factors including: (1) the spatio-temporal heterogeneity of spike’s MEG signal; (2) their signal- to-noise ratio; and (3) the statistical stability of the solution (71). Other modeling approaches have been proposed to overcome this limitation. Our group assesses the volumetric sources of spikes based on a beamforming method, which has been validated using intracranial EEG as a gold standard (Volumetric Imaging of Epileptic Spikes, VIES) (71). Using that method, we found that 8 out of 21 patients had focal sources of spikes while nine others had either lateralized but non-focal or bilateral sources of spikes. In successfully operated patients (i.e., seizure-free post-operatively), the proportion of those with focal sources of spikes on preoperative MEG varies between 71 and 100% (26, 68–70). Chowdhury et al. proposed to use the MEM source localization framework with realistic models based on cortical parcels to localize spatially extended generators (64). That study showed that methods implemented within the MEM framework were sensitive to all spatial extents of the sources ranging from 3 to 30 cm^2^, whatever were the number and size of the parcels defining the model.

The localizing value of MSI to delineate the SOZ has been evaluated in several studies with various gold standards (see Table 2). Those using intracranial EEG have reported high spatial congruence between the center of spiking areas determined with MSI and the SOZ, varying from 81 to 100% (26, 70, 72). The level of spatial overlap between MSI volumes and SOZ is lower due to: (a) spiking volumes determined with MSI are only a statistical model of the true brain spiking volumes and (b) brain spiking areas are usually more widespread than the true SOZ. The accuracy of MSI is higher for focal spiking volumes than for more widespread epileptiform paroxysms, since conventional modeling approaches with current equivalent dipoles are not well adapted to model sources of widespread abnormalities (71). For the latter, alternative methods using distributed sources models or beamforming methods have been proposed, but large-scale clinical validation is still lacking (71). The accuracy of MSI is also higher for superficial cortical sources with tangential orientation (e.g., neocortical areas of the frontal or parietal lobes), than for deep sources with radial orientation.

**Table 2:** **Diagnostic accuracy of MSI in MRI-negative focal epilepsy**.

Study	Lobe	Channels	Number of patients	Inverse model	Patients’ age	Sensitivity	Accuracy
Minassian et al. (73)	TL, ET	74/148	11	SDM	8–16	Focal MEG: 8/11	Congruence MEG/iEEG: 8/8
						Surgery: 5/5	MEG good outcome prediction: 4/8
							Congruence MEG/surgery: NA
RamachandranNair et al. (67)	TL, ET	151	22	SDM	4–18	Focal MEG: 18/22	Congruence MEG/iEEG: NA
						Surgery: 9/9	MEG good outcome prediction: 8/18
							Congruence MEG/surgery: 18/22
Funke et al. (74)	TL, ET	306	17	SDM	3–54	Focal MEG: 6/17	Congruence MEG/iEEG: NA
						Surgery: NA	MEG good outcome prediction: NA
							Congruence MEG/surgery: NA
Seo et al. (26)	TL, ET	275	14	Beamforming	3–18	Focal MEG: 11/14	Congruence MEG/iEEG: 9/11
						Surgery: 6/7	MEG good outcome prediction: 6/9
							Congruence MEG/surgery: 9/11
Heers et al. (75)	ET	74	3	SDM	19–45	Focal MEG: 3/3	Congruence MEG/iEEG: 3/3
						Surgery: 2/2	MEG good outcome prediction: 2/3
							Congruence MEG/surgery: 3/3
Jung et al. (70)	TL, ET	275	21	Beamforming	4–55	Focal MEG: 8/21	Congruence MEG/iEEG: 8/8
						Surgery: 6/6	MEG good outcome prediction: 6/7
							Congruence MEG/surgery: 7/7
Widjaja et al. (76)	TL, ET	151	26	SDM	0.9–17.6	Focal MEG: 19/22	Congruence MEG/iEEG: NA
						Surgery: 14/16	MEG good outcome prediction: 14/18
							Congruence MEG/surgery: 18/22
Wilenius et al. (72)	TL, ET	306	13	SDM	12–47	Focal MEG: 8/13	Congruence MEG/iEEG: 8/8
						Surgery: 5/7	MEG good outcome prediction: 5/8
							Congruence MEG/surgery: 7/22
Wu et al. (69)	TL, ET	74/248	18	SDM	23–60	Focal MEG: 12/18	Congruence MEG/iEEG: NA
						Surgery: 10/10	MEG good outcome prediction: 10/12
							Congruence MEG/surgery: NA

Studies defining SOZ as the resected area in patients seizure-free post-operatively have also reported high accuracy of MSI, between 80 and 100% (26, 68, 70). However, the accuracy of MSI should not be overestimated since only a minority of the single source localizations corresponding to individual spikes are finally included in the surgical resection. For example, Fischer et al. proposed to generate statistical volumes from the sources of individual spikes (77). Using that method, they showed that an average of 20% of the MEG volumes were included in the resection volumes. Moreover, there was a correlation between a favorable outcome and a high coverage of the MEG volumes by the resection volume. For patients with several clusters of spikes, seizure freedom is not directly related to the proportion of clusters of spikes being resected. As a whole, this suggests that MSI is a good indicator of the site of surgical resection, although it is not yet possible to delineate perfectly the cortical regions to be resected based on MSI results only.

Several MEG studies have shown that the presence of a focal spiking source was predictive of surgical success. A recent study showed that the odds-ratio (adjusted for epilepsy and MRI classification) for MSI prediction of seizure-free outcome was 4.4 (23). Tightly clustered spike dipoles were also associated with favorable surgery outcome. Our group recently showed that the extent of the spiking volume determined with MSI is predictive of likelihood of successful localization of the SOZ using intracranial EEG (70). For patients with a focal spiking volume, the SOZ defined by SEEG was clearly localized in all cases and most patients (6/7, 86%) had a good surgical outcome. Conversely, SEEG failed to delineate a SOZ in 57% of patients with a lateralized spiking volume, and in two patients with bilateral spiking volume.

## Combination of PET and MSI Data during Presurgical Evaluation

### Comparative advantages and limitations of PET and MSI in patients with MRI-negative epilepsy

According to the data discussed above, the advantages and limitations of PET imaging and MSI in the assessment of the SOZ in patients with MRI-negative epilepsy appear comparable. Both approaches show variable sensitivity among series, which could be explained by differences in the population studied, selection bias (e.g., operated patients or patients with clearcut spikes on scalp-EEG selected for MEG), and various methods used for data analysis. The presence of a single focal abnormality, either on MEG or PET, is associated with a good surgical outcome. However, the spatial congruence between the volume of PET or MEG abnormalities, and that of the SOZ, is far from perfect in many patients. Overall, the diagnostic accuracy of these investigations often remains insufficient to guide surgical decision without prior intracranial EEG in patients with MRI-negative epilepsy. Conversely, both investigations appear useful to guide the placement of depth or subdural electrodes, and help promoting an optimal sampling of the SOZ by these electrodes. A few MEG studies specifically investigated this issue (22, 78, 79). Knowlton et al. showed that MSI indicated additional electrode coverage in 18 of their 77 patients (not initially planed when MSI results were blinded but added after MSI was unblinded). Thirty-nine percent of those patients had seizures involving the additional electrodes implanted on the basis of MSI findings (78). Similarly, several studies have suggested that PET results might be useful to guide electrode placement and to improve coverage of the SOZ (36, 80).

### Potential diagnostic added value of the combination of PET and MSI data

Considering the diagnostic performance of MSI and PET imaging, an important issue is to evaluate whether multimodal imaging using a combination of the two techniques might improve the presumptive localization of the SOZ, as compared to each technique used in isolation. Only two studies investigated this issue (23, 76). Knowlton and colleagues studied the localizing value of association of MSI and [^18^F]-FDG-PET in 51 patients with MRI-negative epilepsy who had achieved seizure freedom after epilepsy surgery (23). The proportion of patients with localized abnormalities on both MSI and [^18^F]-FDG-PET was low, with a combined sensitivity of 25% (95% CI 15.2–28.0). However, this diagnostic criterion was highly specific: 95% specificity for MSI + PET in comparison with 79% (58.7–92.5) for MSI or PET alone (23). In addition, when associated with intracranial EEG data, the localizing accuracy of [^18^F]-FDG-PET-MSI combination reached 100% (23). The other study included 22 children with MRI-negative epilepsy and showed similar results (76). When both [^18^F]-FDG-PET and MSI demonstrated abnormalities concordant with the cortical resection, specificity for Engel I seizure outcome was 100% (95.7–100) whereas sensitivity of the combination was lower than that of individual tests (55.0%) (76).

### Further research issues

Despite these encouraging results, several issues remain to be addressed. The exact impact of combining MSI and PET on the placement of intracranial electrode, the final surgical decision, and the post-operative outcome has not been specifically investigated. According to the above studies, a positive impact is expected but still needs to be demonstrated and quantified in various populations (e.g., TLE and extra-temporal epilepsy). For instance, the sensitivity and the specificity of [^18^F]-FDG-PET in MRI-negative patients appears higher in TLE than in extra-temporal epilepsy, while the intrinsic characteristics of MSI result in higher accuracy in neocortical epilepsy than in deeply located epileptic foci, such as those responsible for mTLE. Combination of both tests might improve SOZ localization in both situations, but also requires careful interpretation of potential discordances between PET and MSI. In neocortical epilepsy, where [^18^F]-FDG-PET abnormalities are frequently more widespread than the SOZ, the detection of a MEG focus might help to refine the interpretation of PET images by highlighting spatially concordant abnormalities that may be the core of the epileptogenic network. In mesial temporal lobe epilepsy where a MEG focus is not detected or falsely localized outside the mesial temporal structure, second interpretation of MSI results in light of PET data might be useful. Both PET and MEG were found to help detecting subtle MRI abnormalities, and in particular small FCD, in patients previously considered MRI-negative (7, 10, 72, 74, 75). Whether the combination of [^18^F]-FDG-PET and MSI remains useful when one of the two investigations has allowed to identified a MRI-occult FCD also needs to be addressed. The combination of PET-MSI has been studied using [^18^F]-FDG-PET only. As detailed previously, other PET tracers might have greater sensitivity and specificity than [^18^F]-FDG-PET in specific situations (i.e., 5-HT_1A_ receptors PET in TLE). It might thus be worth investigating the combination of MSI with these other tracers.

## Conclusion

Positron emission tomography and MSI have been proposed as valuable tools to help localizing the SOZ prior to intracranial EEG recordings. However, each of these techniques suffers from limitations which might hamper a precise delineation of the SOZ in patients with MRI-negative focal epilepsy. Combination of PET and MEG might increase the sensitivity and specificity relatively to individual diagnostic tests in these patients, an issue which still needs to be formally demonstrated.

## Conflict of Interest Statement

The authors declare that the research was conducted in the absence of any commercial or financial relationships that could be construed as a potential conflict of interest.
